# Selective temporary angioembolisation in older adults with pelvic trauma – Two cases

**DOI:** 10.1016/j.tcr.2025.101248

**Published:** 2025-09-23

**Authors:** Jan Gewiess, Alois Komarek, Drosos Kotelis, Jose Roshardt, Silviya Ivanova, Thomas Lustenberger, Johannes Dominik Bastian

**Affiliations:** a Department of Orthopaedic Surgery and Traumatology, Bern University Hospital, University of Bern, Switzerland; b Department of Diagnostic, Interventional, and Pediatric Radiology, Bern University Hospital, University of Bern, Switzerland; c Department of Vascular Surgery, Bern University Hospital, University of Bern, Switzerland

**Keywords:** Acetabular fracture, Hemorrhage, High-energy, Angioembolization, Corona mortis

## Abstract

Hemorrhage is a major concern in the management of high-energy pelvic and acetabular fractures, with complex acetabular patterns often associated with significant blood loss. While angioembolization is well-established in pelvic trauma, its role in temporarily minimizing intraoperative hemorrhage during acetabular fixation remains underexplored.

This report presents two cases of polytraumatized older adults with combined pelvic ring and complex acetabular fractures managed with preoperative selective, temporary angioembolization. Case 1 involved a 76-year-old male with hemorrhagic shock and multiple injuries, including a left associated both-column (ABC) acetabular fracture and superior gluteal artery bleeding. Case 2 involved a 68-year-old male with a right ABC acetabular fracture and multilevel internal iliac artery injury following an avalanche. Both patients underwent early selective angioembolization with gelatin sponge prior to staged fracture fixation using combined intra- and extrapelvic surgical approaches.

In both cases, cumulative intraoperative blood loss was limited to <1200 mL despite the need for extensive fixation involving multiple surgical windows and approaches. Postoperative recovery was favorable, with both patients regaining independent ambulation and functional hip mobility. No embolization-related complications or surgical site infections were observed.

Preoperative bleeding in acetabular fractures often originates from cancellous bone and arterial branches, including the superior gluteal and obturator arteries. Intraoperative hemorrhage remains a significant challenge, particularly in elderly or physiologically compromised patients.

Selective, temporary angioembolization appears to be a promising adjunct for minimizing intraoperative hemorrhage in high-risk patients undergoing combined pelvic and acetabular fracture fixation. Further studies are warranted to evaluate its efficacy and safety in broader clinical contexts.

## Introduction

Hemorrhage is a well-recognized complication associated with high-energy pelvic and acetabular fractures [1]. In cases of isolated, complex acetabular fractures, preoperative blood loss has been reported to exceed 1300 mL. Furthermore, intraoperative blood loss during surgical fixation of these fractures has been documented to reach or surpass 2000 mL in approximately 33 % of cases [2,3]. In addition to medical and anesthetic management, meticulous surgical technique and the temporary, selective reduction of regional blood flow may contribute to minimizing blood loss in appropriately selected patients. This report presents two cases of polytrauma in older adults with limited physiological reserve involving high-energy mechanisms that resulted in combined pelvic ring and complex acetabular fractures. Both patients underwent preoperative selective, temporary angioembolization as a strategy for intraoperative hemorrhage control.

## Case presentation

### Case 1

**Fig. 1:**
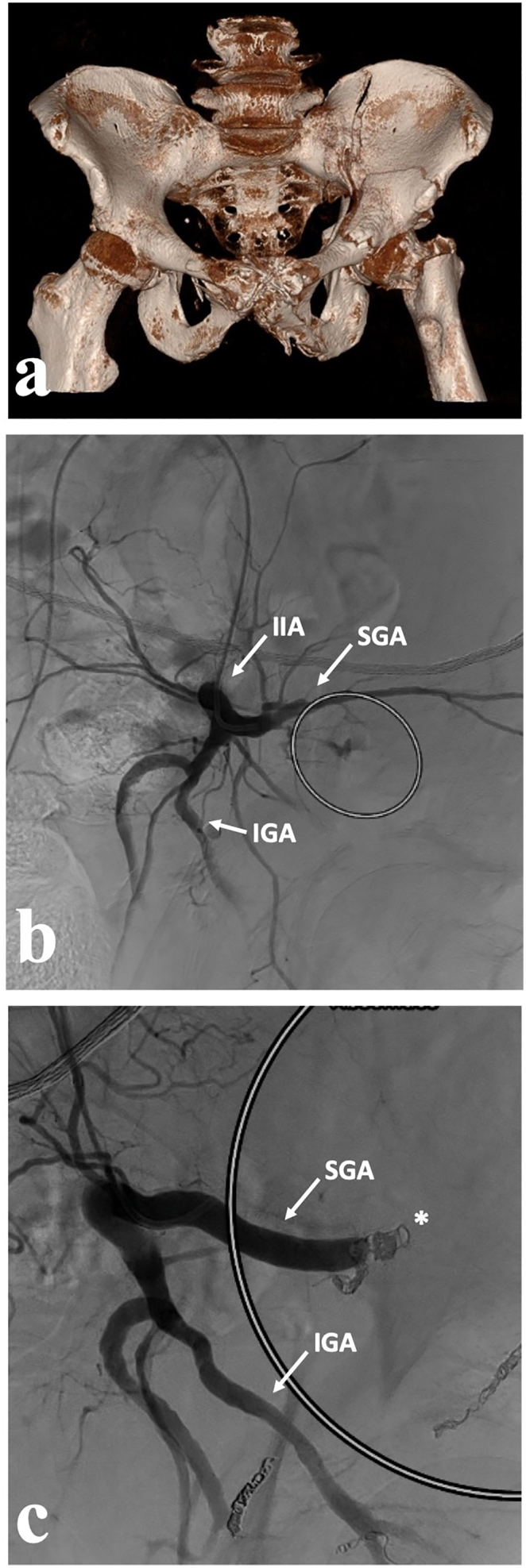
Case 1. (a) 76-year-old male with a Young & Burgess lateral compression type II pelvic ring injury, a left associated both column (ABC) fracture and ipsilateral femoral neck fracture after a 2 m fall from a ladder; (b-c) preoperatively, temporary selective angioembolization of the superior gluteal artery was performed (asterisk in c) as there was active bleeding from the superior gluteal artery (circle in b). IGA – inferior gluteal artery, IIA – internal iliac artery, SGA – superior gluteal artery.

**Fig. 2:**
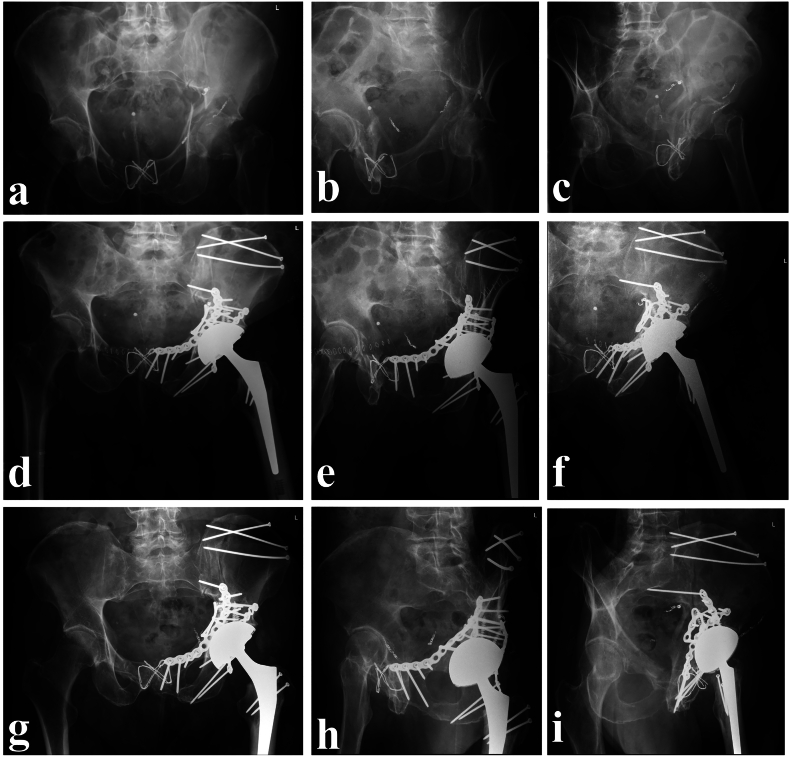
Case 1; (a-c) Pelvis a.-p., obturator, and ala radiographs preoperatively demonstrate the extent of the displacement of the quadrilateral plate and medialization of the femoral head and the femoral neck fracture; (d-f) in a staged procedure, ORIF of the acetabulum and hip arthroplasty were performed at two and nine days post trauma; (g-i) at the six month follow-up, the patient was ambulating without crutches, living independently at home and without the need for analgesics.

A 76-year-old male with a history of hypertensive heart disease and arrhythmogenic cardiopathy on rivaroxaban presented to the shock room in hemorrhagic shock following a 2-m fall from a ladder. Initial resuscitation included the administration of 5 L of Ringer's solution, 1 g of tranexamic acid, 4000 international units of prothrombin complex concentrate, 1 g of fibrinogen, 2 erythrocyte concentrates, and 4 units of fresh frozen plasma. Subsequent whole-body CT revealed a Young & Burgess lateral compression type II pelvic ring injury, a left associated both-column (ABC) acetabular fracture, active bleeding from the superior gluteal artery, an ipsilateral displaced femoral neck fracture, an ipsilateral open olecranon fracture, and multiple ipsilateral rib fractures (ISS 38). The patient underwent selective temporary angioembolization of the superior gluteal artery using gelfoam (Fig. 1). A staged surgical procedure was planned for pelvic fracture fixation; two days post-injury, open reduction and internal fixation of the anterior pelvic ring was performed using a suprapectineal plate via the Stoppa approach and the first ilioinguinal window. Estimated intraoperative blood loss was 500 mL. Nine days post-trauma, osteosynthesis of the posterior column was completed and hip arthroplasty was performed using a lateral approach with a trochanteric osteotomy (Fig. 2). Estimated intraoperative blood loss was 700 mL. The patient was discharged to a nursing facility with non-weight bearing restrictions 28 days after the trauma. At six weeks postoperatively, he presented in a wheelchair in a deconditioned state; subsequently, full weight bearing was permitted and he was transferred to a rehabilitation center. At the six-month follow-up, the patient was ambulating without crutches, living independently at home and without the need for analgesics, demonstrating left hip range of motion of flexion/extension of 90–0-0°, internal/external rotation of 30–0-60°, along with an M4- of the hip abductors.

### Case 2

A 68-year-old male with no significant past medical history presented to a peripheral hospital with pulseless electrical activity and required cardiopulmonary resuscitation following an avalanche accident. Return of spontaneous circulation was achieved within two minutes, with no reported downtime. Whole-body computed tomography (CT) demonstrated a Young & Burgess lateral compression type II pelvic ring injury, a right-sided associated both column (ABC) acetabular fracture, multilevel traumatic dissection of the right internal iliac artery, and a concomitant lesion of the right internal iliac vein. Additional findings included a right-sided tension pneumothorax, nondisplaced fractures of the right ribs, and hypothermia with a core temperature of 31.7 °C (ISS 57). An emergent tube thoracostomy was performed, followed by exploratory laparotomy, vascular repair, and preperitoneal pelvic packing due to ongoing hemodynamic instability. On post-injury day two, the patient underwent surgical depacking and definitive abdominal closure.

**Fig. 3:**
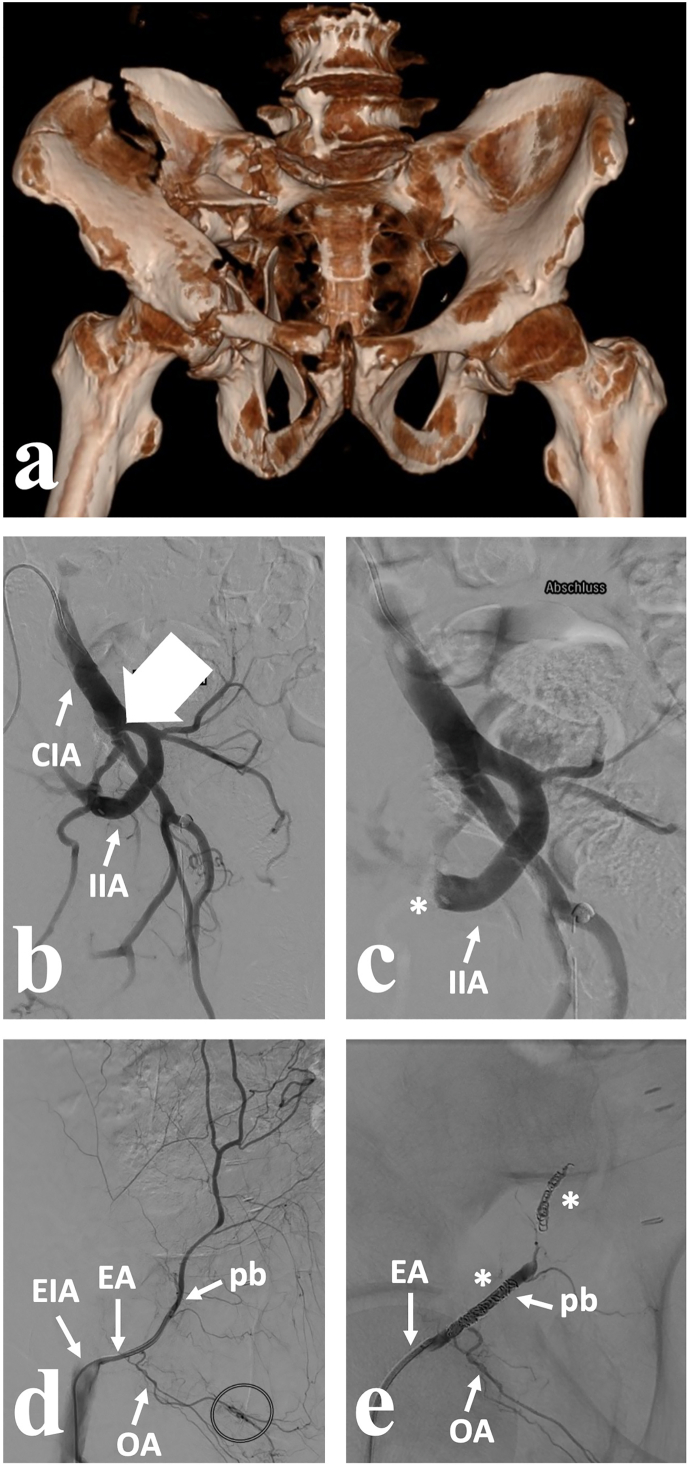
Case 2. (a) 68-year-old male with a Young & Burgess lateral compression type II pelvic ring injury, a right-sided associated both column (ABC) acetabular fracture, multilevel traumatic dissection (big arrow in b) of the right internal iliac artery, and a concomitant lesion of the right internal iliac vein after an avalanche accident; 4 days post-injury, protective temporary angioembolization of the right internal iliac artery (asterisk in c) and selective embolization of the pubic branch of the inferior epigastric artery (asterisks in e) were performed. CIA – common iliac artery, EA – epigastric artery, EIA – external iliac artery, IIA – internal iliac artery, OA – obturator artery, pb – pubic branch.

**Fig. 4:**
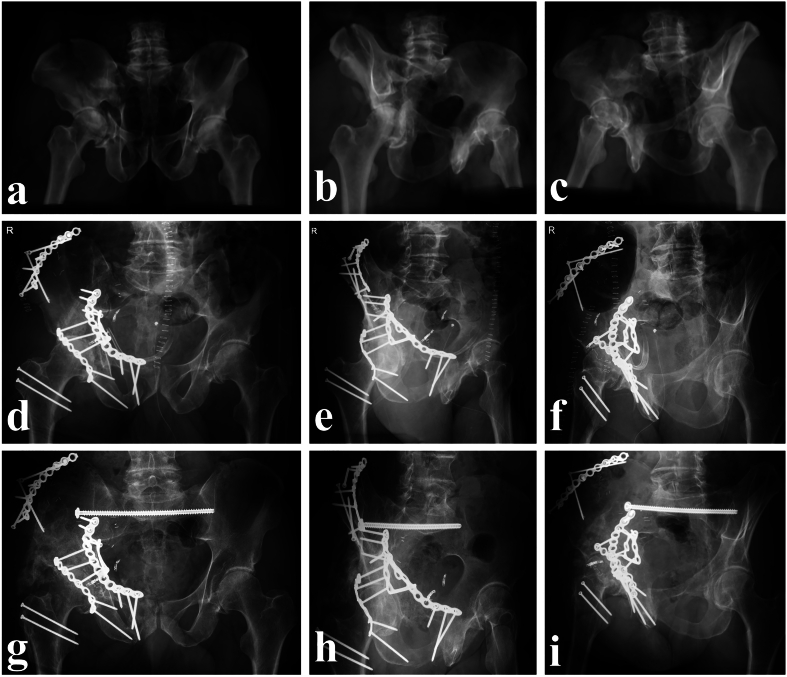
Case 2; (a-c) 3D reconstructions of pelvis a.-p., obturator, and ala projections preoperatively demonstrate the extent of the displacement of the quadrilateral plate and medialization of the femoral head; (d-f) five and eight days post-injury, open reduction and internal fixation of the acetabular fracture via *Re*-laparotomy, an extended first ilioinguinal window combined with surgical hip dislocation in the floppy lateral position and transiliac–transsacral screw fixation of the posterior pelvic ring were performed; (g-i) at the three month follow-up, the patient was ambulatory with crutches and reported no pelvic pain despite some degree of heterotopic ossification.

On post-injury day four, the patient was transferred to a tertiary care center for definitive management of the pelvic injuries. Selective, temporary angioembolization of the right internal iliac artery was performed using gelfoam, and selective embolization of the pubic branch of the inferior epigastric artery was achieved using metallic coils (Fig. 3). On post-injury day five, the patient underwent open reduction and internal fixation of the acetabular fracture via the inferior laparotomy (modified Stoppa), the first ilioinguinal window combined with surgical hip dislocation in the floppy lateral position (Fig. 4). Estimated intraoperative blood loss was 1100 mL. Percutaneous transiliac–transsacral screw fixation of the posterior pelvic ring was performed. The patient was discharged to a rehabilitation facility on day 17. At the three-month follow-up, the patient was ambulatory with crutches and reported no pelvic pain. Clinical examination revealed grade M4 of the hip abductors and right hip flexion/extension 90–0-0° and internal/external rotation 10–0-40°.

## Discussion

Acetabular fractures accompanied by injuries to the pelvic ring and proximal femur frequently necessitate extensive and multiple surgical approaches. Both cases presented herein required combined intra- and extrapelvic surgical approaches to achieve adequate fracture stabilization. A “pelvic ring first” approach is advocated for mechanically connected pelvic and acetabular fractures (Case 1), whereas an “acetabular fracture first” strategy may be preferable in cases of relatively stable posterior pelvic ring injuries where anatomical reduction is achievable (Case 2) [4].

Although hemorrhage is a well-recognized concern in pelvic fractures, its significance is frequently underestimated in acetabular fractures. A retrospective analysis of nearly 600 patients demonstrated that complex acetabular fracture patterns—specifically associated both-column, T-type, and anterior column posterior hemitransverse fractures—are significantly associated with preoperative blood loss exceeding 1300 mL^2^. Additionally, a positive linear correlation was observed between time to surgery and the volume of preoperative hemorrhage.

Preoperative bleeding in acetabular fractures primarily originates from exposed cancellous bone and branches of the external and internal iliac arteries and their anastomoses. The superior gluteal, obturator, and lateral sacral arteries represent the most common arterial sources of hemorrhage [5]. Notably, multiphase postmortem CT angiography has identified a significant association between acetabular fractures and bleeding from the obturator artery.

As opposed to preoperative hemorrhage, intraoperative bleeding is recognized as a major challenge in acetabular fracture fixation. Fixation via anterior approaches has been reported to result in an average blood loss ranging from 100 to 1500 mL [[6], [7], [8]]. Surgical hip dislocation for acetabular fracture management yields an average intraoperative blood loss of approximately 700 mL [9]. Selected patients, such as older adults suffering high-energy and/or polytrauma with limited physiological reserve suffering significant comorbidities, taking anticoagulants and starting into surgery (with estimated longer operating time, e.g. multiple approaches) with low hemoglobin levels may benefit from more extended measures for intraoperative hemorrhage control.

Angioembolization performed early in the course of management demonstrates a success rate of 74–100 % in controlling pelvic hemorrhage [10]. The most commonly embolized vessels include the internal iliac artery (67 %), unnamed branches of the internal iliac artery (17 %), superior gluteal artery (4 %), obturator artery (4 %), and internal pudendal artery (3 %). In a retrospective review by Manson et al. from 2013, a 58 % infection rate was reported in 12 patients undergoing selective angioembolization prior to acetabular fracture fixation [11]. However, this study did not report perioperative blood loss. Surgical site infections following acetabular fracture fixation have been linked to prolonged operative times, elevated body mass index, extended intensive care unit stays, higher volumes of transfused packed red blood cells, and associated genitourinary or abdominal trauma [12].

Considering the broad definition of deep infection (requiring return to the operating room for debridement within one year), and the lack of information on confounding factors such as patient comorbidities, concomitant injuries, and surgical duration, it is challenging to infer a direct causal link between angioembolization and deep infection based on the data presented by Manson et al. Interestingly, four of the seven patients who developed infection had undergone complete embolization of the entire internal iliac artery. Specific or temporary embolization techniques were not described. In the presented cases, selective temporary angioembolization was performed using gelatin sponge torpedoes, which typically resorb within seven days and are intended to reduce blood supply to the fracture site while preserving perfusion through arterial branches. Despite the complexity of the reconstructive procedures, which involved multiple approaches to both pelvic and acetabular fractures with an anticipated intraoperative blood loss of more than 2000 mL, cumulative intraoperative blood loss was limited to less than 1200 mL in both patients.

To our knowledge, no prior studies have evaluated temporary selective angioembolization as an adjunct to minimize intraoperative blood loss and facilitate perioperative management during combined pelvic acetabular fracture fixation in selected patients at high risk for intraoperative complications related to hemorrhage.

## Conclusion

This case report demonstrates that selective, temporary angioembolization can be a safe and effective preoperative intervention for minimizing both pre- and intraoperative hemorrhage. It should be considered a valuable adjunct in the management of patients with combined acetabular and pelvic fractures who are at high risk for significant intraoperative bleeding especially if assumed to originate from cancellous bone.

## CRediT authorship contribution statement

**Jan Gewiess:** Writing – review & editing, Writing – original draft, Visualization, Validation, Supervision, Software, Resources, Project administration, Methodology, Investigation, Formal analysis, Data curation, Conceptualization. **Alois Komarek:** Writing – review & editing, Validation, Supervision, Project administration, Methodology, Investigation, Conceptualization. **Drosos Kotelis:** Writing – review & editing, Validation, Supervision, Project administration, Conceptualization. **Jose Roshardt:** Writing – review & editing, Visualization, Validation, Methodology. **Silviya Ivanova:** Writing – review & editing, Visualization, Validation, Methodology. **Thomas Lustenberger:** Writing – review & editing, Visualization, Validation, Supervision, Project administration, Methodology. **Johannes Dominik Bastian:** Writing – review & editing, Visualization, Validation, Supervision, Software, Resources, Project administration, Methodology, Investigation, Formal analysis, Data curation, Conceptualization.

## Declaration of competing interest

The authors declare that they have no known competing financial interests or personal relationships that could have appeared to influence the work reported in this paper.
